# Polymorphisms of Dectin-1 and TLR2 Predispose to Invasive Fungal Disease in Patients with Acute Myeloid Leukemia

**DOI:** 10.1371/journal.pone.0150632

**Published:** 2016-03-10

**Authors:** Mike Fischer, Baerbel Spies-Weisshart, Karin Schrenk, Bernd Gruhn, Susan Wittig, Anita Glaser, Andreas Hochhaus, Sebastian Scholl, Ulf Schnetzke

**Affiliations:** 1 Klinik für Innere Medizin II, Abteilung für Hämatologie und Internistische Onkologie, Universitätsklinikum Jena, Jena, Germany; 2 Klinik für Kinder- und Jugendmedizin, Universitätsklinikum Jena, Jena, Germany; 3 Institut für Humangenetik, Universitätsklinikum Jena, Jena, Germany; Queen's University Belfast, UNITED KINGDOM

## Abstract

**Background:**

Patients with acute myeloid leukemia (AML) who undergo induction chemotherapy are at high risk for invasive fungal disease (IFD). Dectin-1, a C-type lectin family member represents one of the most important pattern recognition receptors of the innate immune system and single nucleotide polymorphisms (SNPs) in the Dectin-1 gene have been associated with an increased risk of infectious complications. We sought to investigate the impact of three different Dectin-1 SNPs and one TLR2 SNP on developing IFD in 186 adult patients with newly diagnosed AML following anthracycline-based induction chemotherapy.

**Patients and methods:**

Genotyping of Dectin-1 SNPs (rs16910526, rs3901533 and rs7309123) and TLR2 SNP (rs5743708) was performed by TaqMan method and pyrosequencing. IFD was defined according to the EORTC/MSG consensus guidelines. Multiple logistic regression analyses were applied to evaluate the association between the polymorphisms and the occurrence of pulmonary infections. Dectin-1 expression studies with SNP genotyped human monocytes were performed to elucidate susceptibility to IFD following chemotherapy.

**Results:**

We could demonstrate that patients carrying the Dectin-1 SNP rs7309123 G/G (n = 47) or G/G and C/G (n = 133) genotype revealed a significant higher risk for developing both pneumonia in general (adjusted odds ratio (OR): 2.5; p = 0.014 and OR: 3.0, p = 0.004) and pulmonary IFD (OR: 2.6; p = 0.012 and OR: 2.4, p = 0.041, respectively). Patients carrying the TLR2 SNP rs5743708 (R753Q, GA/AA genotype, n = 12) also revealed a significantly higher susceptibility to pneumonia including IFD. Furthermore, Dectin-1 mRNA expression in human monocytes was lower following chemotherapy.

**Conclusion:**

To our best knowledge, this study represents the first analysis demonstrating that harbouring polymorphisms of Dectin-1 (rs7309123) or TLR2 (rs5743708) represents an independent risk factor of developing IFD in patients with AML undergoing induction chemotherapy.

## Introduction

Patients with acute myeloid leukemia (AML) who undergo curative intent induction chemotherapy are at high risk for infectious complications. Despite advances in prophylaxis against bacterial and fungal pathogens infections remain a major cause of morbidity and mortality during long lasting neutropenia in this group of patients [1, 2].

Invasive aspergillosis (IA) is the most common invasive fungal infection during induction chemotherapy and remains a life-threatening condition. Real-life data of invasive fungal disease (IFD) according to EORTC/MSG criteria in AML patients treated with induction chemotherapy describe an incidence of up to 27% when possible IFD is considered as well [3–5].

After the recognition of fungal conidia the innate immune system acts by multiple interactions of many receptors at several sites. Neutrophils and monocytes are activated by pattern recognition receptors (PRRs) recognizing fungal pathogens. Dectin-1 (Dendritic cell-associated C-type lectin-1), a C-type lectin family member represents one of the most important and most studied PRRs in the innate immune response against *Aspergillus spp*. Dectin-1 is highly expressed on neutrophils, monocytes and dendritic cells (DCs) [6]. Recently, Dectin-1 expression was also found on pulmonary epithelial cells, emphasizing the importance of Dectin-1 and its potential role in the pathogenesis of pulmonary IFD [7]. Dectin-1 recognizes fungal ß-glucans, induces phagocytosis and the production of various soluble mediators for clearance of the fungal pathogen. In addition, the adaptive immune system is also directly modulated by Dectin-1 in a Th1 and Th17 dependent manner [8].

Unlike other non-Toll-like receptors (TLRs) PRRs Dectin-1 is characterised by the presence of a functional, tyrosine-based activation like motif (ITAM) at its cytoplasmic tail. Downstream signalling of Dectin-1 is partially mediated through the tyrosine kinase Syk which directly interacts with the phosphorylated receptor via its SH2 domains [9].

Polymorphisms in human PRRs have been associated with an increased risk of infectious complications including IA in susceptible hosts [10]. A functional single nucleotide polymorphism (SNP) in the Dectin-1 gene (Y238X—rs16910526) generates an early stop codon leading to the loss of the last C-terminal 10 amino acids of the carbohydrate-recognition domain resulting in a diminished surface expression of the Dectin-1 receptor on immune cells [11]. Two other polymorphisms in Dectin-1 introns (rs3901533, rs7309123) were also associated with the development of IA in patients with hematologic diseases although the functional consequences have not been studied yet [12].

Recently, we could demonstrate that functionally relevant TLR2 (Arg753Gln—rs5743708) and TLR4 (Asp299Gly—rs4986790, Thr399Ile—rs4986791) polymorphisms significantly contribute to infectious complications like sepsis and pneumonia in AML patients undergoing induction chemotherapy [3]. There are numerous reports in the literature that describe a synergism between Dectin-1 and TLR2 receptors with regard to the release of cytokines and crosstalk signalling in response to fungal pathogens [13]. Although TLR2 has been intensively studied for its significance on bacterial lipoprotein signalling it has also been shown that the receptor plays a role in host defense against fungal infections both alone and synergistic with Dectin-1 [14].

In this study we hypothesized an impact of three different Dectin-1 SNPs and one functionally relevant TLR2 SNP on the susceptibility to IFD in 186 adult AML patients who received and anthracycline-based induction chemotherapy.

## Patients and Methods

### Patients

A group of 186 Caucasian patients (83 male, 103 female; median age 58 years, range 19–78 years) with newly diagnosed AML (excluding acute promyelocytic leukemia) were included in this retrospective single institution study covering diagnoses between 2000 and 2014. Informed written consent for all determinations and genetic analyses was obtained from all participants in accordance with the Declaration of Helsinki. The local ethics committee (Ethikkommisson, Universitätsklinikum Jena, Germany) provided institutional review board approval for this study.

Treatment protocols for induction chemotherapy were applied according to the Ostdeutsche Studiengruppe für Hämatologie und Onkologie (OSHO): AML96 or AML2002 protocols containing idarubicine for patients up to 60 years old and AML97 or AML2004 protocols containing mitoxantrone for elderly patients [15, 16].

All patients received either trimethoprim–sulfamethoxazole or ciprofloxacin and fluconazole or posaconazole for antibiotic and antifungal prophylaxis, respectively.

Clinical data are presented in Table 1. The observational period was defined by the interval between start of induction chemotherapy and the discharge from hospital after hematologic reconstitution.

**Table 1:** Patients and clinical characteristics.

	n = 186	P values *
Median age, years (range)	58 (19–78)	n.s.
Male sex, no (%)	83 (44.6)	n.s.
Cytogenetic risk group		n.s.
good (%)	20 (10.7)	
intermediate (%)	108 (58.1)	
poor (%)	58 (31.2)	
WBC count at diagnosis (/nl), median (range)	14.2 (0.3–330)	n.s.
Platelet count at diagnosis (/nl), median (range)	45 (2–332)	n.s.
Hemoglobin level at diagnosis (mmol/l), median (range)	5.7 (2.5–8.8)	n.s.
Peripheral blood blasts (%), median (range)	27 (0–99)	n.s.
Bone marrow blasts (%), median (range)	75 (17–99)	n.s.
Outcome after induction chemotherapy		
Alive	183	
Dead	3	

* P-values (calculated for IFD).

Abbreviation

WBC, white blood cell.

Following induction chemotherapy three patients died of infectious complications including two within the first 30 days. The third patient did not show hematologic recovery and died at day 84 from the start of induction chemotherapy without evidence of relapse.

### Cytogenetic analyses

Bone marrow or peripheral blood cells were karyotyped according to the International System for Human Cytogenetic Nomenclature [17]. Determination of cytogenetic risk groups was performed according to established recommendations [18]. Due to poor quality material cytogenetic analysis could not be performed in six patients.

### Diagnostic criteria of pneumonia

Pneumonia was defined as a new infiltrate on chest radiograph (X-ray and/ or computer tomography) in combination with at least two of the following criteria: cough, sputum production, temperature >38°C or <35°C, hemoptysis, thoracic pain or auscultatory findings consistent with pneumonia. Pneumonia was classified as atypical by radiographic criteria and pulmonary IFD was diagnosed based upon the criteria reported by the European Organization for Research and Treatment of Cancer/Invasive Fungal Infections Cooperative Group and the National Institute of Allergy and Infectious Diseases Mycoses Study Group (EORTC/MSG) in 2008 [19].

### Analysis of TLR and Dectin-1 polymorphisms

Genotyping of the TLR2 R753Q rs5743708 and Dectin-1 Y238X rs16910526 was performed by pyrosequencing. The genotype of Dectin-1 rs7309123 and rs3901533 was determined by TaqMan assay. Genomic DNA was extracted from peripheral blood or bone marrow samples using the QIAamp Blood Mini Kit according to the manufacturer´s instructions (Qiagen, Hilden, Germany). PCR and sequencing primers were designed using the PSQ assay design tool: TLR2 rs5743708 forward primer: 3´-Biotin- GGTGCAAGTATGAACTGGACTTCT-5´, reverse primer 3´-GGCCACTCCAGGTAGGTCTT-5´; Dectin-1 rs16910526 forward primer: 3’- TGACTGACACGTGAATCCATACA-5’, reverse primer: 3’- Biotin- TCAATGTAAGAGGAAGGGTGGAG -5’. 100 ng genomic DNA was amplified using the following cycling conditions for the TLR2 rs5743708: 95°C for 5 minutes, 35 cycles of 92°C for 30 seconds, 63°C for 30 seconds and 72°C for 30s followed by a final extension of 72°C for 5 minutes. For the Dectin-1 rs16910526 PCR annealing temperature was 62°C.

For all PCRs primer concentrations were 600 nM each at a final volume of 25 μl with 1.5 mM MgCl_2_. Pyrosequencing was performed using PyroMark Gold Q96 Reagents with the sequence analysis mode of the PyroMark Q96 ID system according to the manufactorer´s instructions (Qiagen, Hilden, Germany).

Sequencing primers (TLR2 rs5743708: 3´- TCTTGGTGTTCATTATCTTC-´5; Dectin-1 rs16910526: 3´-GAGGGCACACTACACA–´5) were used at 400 nM final concentration.

Allele detection of Dectin-1 rs7309123 und rs3901533 were examined with the use of a commercially available TaqMan SNP genotyping assay kit and PCR System 9700HT (Applied Biosystems, Foster City, CA, USA), in accordance with the manufacturers instructions. TaqMan Genotyper Software (Applied Biosystems, Darmstadt, Germany) was used for genotyping analysis.

Negative and positive controls were included in pyrosquencing and TaqMan assays as a quality control measure.

### Isolation of human monocytes

Peripheral blood samples were collected from AML patients and mononuclear cells (PBMCs) were isolated by density gradient centrifugation using Ficoll-Paque. PBMCs were washed 2x in PBS and subsequently isolated with CD14 Microbeads according to the manufacturer’s instruction (Miltenyi, Bergisch Gladbach, Germany). Monocyte purity was greater than 97% as assessed by flow cytometry (data not shown).

### RNA preparation and qRT-PCR analyses

Total RNA were isolated using innuPREP RNA Mini Kit (Analytik Jena, Jena, Germany) according to standard protocol. First-strand cDNA synthesis was performed with 1 μg of total RNA using M-MLV reverse transcriptase according to standard protocol (Invitrogen, Karlsruhe, Germany). Analyses were performed using the Mastercycler® ep realplex Real-time PCR System (Eppendorf, Hamburg, Germany). The reaction set up (20 μl) was as follows: 20 ng cDNA, 0.5 μM of each primer and 1x FastStart SYBR Green Master (Roche, Mannheim, Germany). The following primer sets were used: Dectin-1 5’-TGCTATATCTATTCAGGGGCTCT-3’ and 5’-GCAGCACACGATCCTTTCTC-3’; RPL13A: 5’- CGGACCGTGCGAGGTAT -3’ and 5’-ACACCTTGAGACGGTCCAGA-3’. All reactions were run at 95°C for 10 min following 40 cycles at 95°C for 10 s, 58°C for 15 s and 72°C for 20 s. Data were analyzed using the 2^- ΔΔCt^ method (Livak and Schmittgen, 2001).

### Dectin-1 surface expression by flow cytometry

5 x 10^5^ cells were washed twice in 1 ml cold PBS containing 0.5% BSA following incubation with Anti-Dectin-1-antibody (Anti-Dectin-1-PE (FAB1859P) and IgG2b-PE isotype control (IC0041P) from R&D-Systems (Wiesbaden, Germany) in FACS tubes (20 min, 4°C) were used. Cells were washed in 3 ml PBS and finally suspended in 500 μl PBS. measured by flow cytometry using a FACSCalibur using Cell Quest^TM^ software (Becton-Dickinson, Heidelberg, Germany). Signals were averaged using the geometric mean and defined as mean fluorescence intensity (MFI). An isotype control IgG staining for each sample was performed and the signal subtracted from the sample.

### Statistics

Odds ratios (OR) and 95% confidence intervals (CI) were calculated using multiple logistic regression analyses. Analyses of risk associations for different polymorphisms were adjusted for sex, cytogenetic risk group (low, intermediate, high), age at diagnosis, white blood count, platelet count and hemoglobin level. Quantitative characteristics were expressed by their median value. Student’s t-test (parametric, two-tailed) and chi-square test were used to identify statistical differences when appropriate. The level of significance was considered to be statistically significant with a p value of ≤ 0.05. All analyses were conducted using the SPSS software package, version 22 (SPSS, Chicago, IL, USA).

## Results

### Frequency of TLR2 and Dectin-1 polymorphisms

Genotyping of the polymorphisms (TLR2 R753Q—rs5743708; Dectin-1 Y238X –rs16910526, Dectin-1 –rs3901533; Dectin-1 –rs7309123) in 186 adult AML patients revealed different frequencies for these investigated SNPs (S1 Fig). In detail, 12 patients (6.5%) were carriers of the TLR2 R753Q polymorphism. Furthermore, in 19 patients (10.2%) the Dectin-1 Y238X SNP was found. The Dectin-1 SNP rs3901533 T/T genotype could only be detected in six patients (3.2%). Due to the very low frequency of this polymorphism it was excluded from further analysis. In contrast, the Dectin-1 polymorphism rs7309123 G/G genotype was found in 47 of 186 patients (25.3%) and 133 patients (71.5%) were carrier of the genotypes C/G or GG.

All genotype frequencies fulfilled the criteria of Hardy-Weinberg equilibrium (https://ihg.gsf.de/ihg/snps.html).

### Occurrence of pneumonia following AML induction chemotherapy

Table 2 summarizes the frequency of different subsets of pneumonia as observed in the cohort of 186 AML patients who received intensive anthracyclin-containing chemotherapy. In detail, 69 patients (37%) developed pneumonia while the majority of these patients fulfilled the criteria of atypical pneumonia as it could be found in 58 of 186 patients (31%).

**Table 2:** Subtypes of pneumonia and its SNP-dependent distribution (%).

	all patients n = 186	TLR2 R753Q rs5743708 n = 12	Dectin-1 Y238X rs16910526 n = 19	Dectin-1 Intron rs7309123 (G/G genotype) n = 47	Dectin-1 Intronrs7309123 (C/G + G/G genotype) n = 133
Pneumonia (including atypical pneumonia)	69 (37%)	10 (83%)	6 (32%)	25 (53%)	58 (44%)
Atypical pneumonia (including pulmonary IFD)	58 (31%)	10 (83%)	6 (32%)	21 (45%)	48 (36%)
Pulmonary IFD*	48 (26%)	7 (58%)	4 (21%)	19 (40%)	40 (30%)
Pulmonary IFD**	9 (5%)	2 (17%)	0 (0%)	5 (11%)	9 (7%)

* including possible, probable and proven IFD according to the EORTC/MSG criteria

** including only probable and proven IFD according to the EORTC/MSG criteria.

The analysis of pulmonary fungal infections according to the EORTC/MSG criteria revealed 48 patients (26%) with pulmonary IFD. In detail, possible, probable and proven IFD could be demonstrated in 39, 7 and 2 patients, respectively. Importantly, in two cases mucormycosis was found and confirmed histologically.

### Impact of TLR2 and Dectin-1 polymorphisms on occurrence of pneumonia

Table 2 also demonstrates the frequencies of different subsets of pneumonia dependent on the presence of the TLR2 or one of the investigated Dectin-1 polymorphisms. In detail, 10 out of 12 patients (83%) harboring the TLR2 R753Q SNP developed atypical pneumonia and 7 out of 12 (58%) fulfilled the criteria of IFD (possible, probable or proven). The frequencies of the different types of pneumonia were as follows for patients carrying either genotype G/G or G/G + G/C of the Dectin-1 polymorphism rs7309123: general pneumonia 53% or 44%, atypical pneumonia 45% or 36% and IFD 40% or 30%, respectively.

Multiple logistic regression analysis was performed for the occurrence of developing distinct subsets of pneumonia including pulmonary IFD according to the EORTC/MSG consensus guidelines (Table 3). Patients harboring either the TLR2 R753Q or the Dectin-1 rs7309123 G/G and G/G + C/G genotype were identified to have a significant higher risk for developing pneumonia in general, as well as atypical pneumonia and even pulmonary IFD. None of the clinical characteristics provided in Table 1 remained statistically significant in this multivariate approach (Table 1).

**Table 3:** Multivariate analyses of the attributable risk of the TLR2 and Dectin-1 polymorphisms.

Genetic variable	*Pneumonia %*	OR (95% CI) *P value*	*Atypical Pneumonia %*	OR (95% CI) *P value*	PulmonaryIFD* *%*	OR (95% CI) *P value*
TLR2 wild type vs.	34%	9.8	28%	13.1	24%	4.5
TLR2 R753Q	83%	(2.1–45.9)	83%	(2.8–62.1)	58%	(1.4–15.1)
rs5743708		0.001		0.001		0.014
Dectin-1 wild type** vs.	38%	1.0	31%	0.8	26%	0.7
Dectin-1 Y238X	32%	n.s.	32%	n.s.	21%	n.s.
rs16910526						
Dectin-1 C/C + C/G vs.	32%	2.5	27%	2.2	21%	2.6
Dectin-1 rs7309123	53%	(1.2–4.8)	45%	(1.1–4.4)	40%	(1.3–5.3)
G/G genotype		0.014		0.028		0.012
Dectin-1 C/C vs.	21%	3.0	19%	2.4	15%	2.4
Dectin-1 rs7309123	44%	(1.4–6.2)	36%	(1.1–5.3)	30%	(1.1–5.6)
G/G + C/G genotype		0.004		0.023		0.041

* including possible, probable and proven IFD according to the EORTC/MSG criteria

** Dectin-1 wild type was defined as negativity of the Y238X allele.

Abbreviations

OR, odds ratio

CI, confidence interval.

In detail, in the TLR2 wild type group pneumonia was documented in 34%, whereas for patients carrying the TLR2 polymorphism the incidence was 83% (OR 9.8; 95% CI 2.1–45.9, *p = 0*.*001)*. The presence of the TLR2 R753Q SNP was also associated with a significantly higher risk for developing atypical pneumonia including pulmonary IFD (OR 13.1; 95% CI 2.8–62.1, *p = 0*.*001* and OR 4.5, 95% CI 1.4–15.1, *p = 0*.*014*, respectively) (Table 3).

Carriers of the G-allele of the Dectin-1 rs7309123 polymorphism (G/G = 47 and G/G + G/C = 133) revealed a strong association with the risk of developing atypical pneumonia and

pulmonary IFD. In detail, pneumonia was diagnosed in 53% of patients harboring the G/G genotype but only in 32% of patients carrying either C/C or C/G genotypes (OR 2.5; 95% CI 1.2–4.8, *p = 0*.*014*). Furthermore, G/G genotype of Dectin-1 rs7309123 showed a strong association with risk of atypical pneumonia and pulmonary IFD (45% vs. 27% and 40% vs. 21%). By multiple logistic regression analysis the Dectin-1 rs7309123 G/G genotype represents an independent risk factor for developing atypical pneumonia (OR 2.2; 95% CI 1.1–4.4, *p = 0*.*028*) and pulmonary IFD (OR 2.6; 95% CI 1.3–5.3, *p = 0*.*012)* (Table 3). A significant correlation was also observed by comparing G/G + C/G genotype vs. C/C genotype of the Dectin-1 rs7309123 polymorphisms (pneumonia: OR 3.0; 95% CI 1.4–6.2, p = 0.004; atypical pneumonia OR 2.4; 95% CI 1.1–5.3, p = 0.023 and pulmonary IFD: OR 2.4; 95% CI 1.1–5.6, p = 0.041) (Table 3).

We also provide data for probable and proven IFD only, although we are aware that these results are based on a rather low number of patients (S1 Table). Importantly, the G/G genotype of the Dectin-1 rs7309123 SNP is also significantly associated with the risk of probable and proven IFD. Interestingly, both patients diagnosed with proven IFDs were carriers of the G/G genotype.

We also analyzed the functionally relevant Dectin-1 polymorphism rs16910526 encoding an early stop codon at position 238 (Y238X). There was no correlation between the presence of that stop codon SNP and the occurrence of pneumonia in general (OR 0.8; 95% CI 0.3–2.2, *p = 0*.*63*). Furthermore, Dectin-1 Y238X did also not affect the frequency of atypical pneumonia (OR 1.1; 95% CI 0.4–3.0, *p = 0*.*9*) or pulmonary IFD (OR 0.7; 95% CI 0.2–2.5, *p = 0*.*65*). Thus, there was no association between the stop-coding SNP of Dectin-1 and the occurrence of pneumonia following AML induction chemotherapy.

### Cluster analysis of TLR2 and Dectin-1 rs7309123 polymorphisms

Because of the functional relationship between TLR2 and Dectin-1 we next analyzed if a clustering of AML patients tested positive for the TLR2 R753Q polymorphism or carrying the Dectin-1 SNP rs7309123 G/G genotype might affect the frequency of pneumonia. Table 4 demonstrates the occurrence of pneumonia in general, atypical pneumonia and pulmonary IFD dependent on the carrier status of the different polymorphisms (TLR2 R753Q + Dectin-1 rs7309123 G/G genotype vs TLR2 wild type + Dectin-1 rs7309123 C/C + C/G genotype).

**Table 4:** Subgroup analysis for TLR2 R753Q and Dectin-1 rs7309123 G/G genotype regarding susceptibility to pneumonia.

Genetic variable	*Pneumonia %*	OR (95% CI) *P value*	*Atypical Pneumonia %*	OR (95% CI) *P value*	Pulmonary IFD* *%*	OR (95% CI) *P value*
Cohort 1						
TLR2 R753Q or	56%	3.1	48%	2.9	42%	3.1
Dectin-1 rs7309123		(1.6–6.1)		(1.5–5.7)		(1.5–6.3)
G/G genotype		*0*.*001*		*0*.*003*		*0*.*002*
Cohort 2						
TLR2 wild type	30%		25%		19%	
Dectin-1 rs7309123						
C/C + C/G genotype						

Abbreviations

OR, odds ratio

CI, confidence interval

* including possible, probable and proven IFD according to the EORTC/MSG criteria.

We can demonstrate that the relative risk for developing general, atypical pneumonia or IFD within that functionally related subgroup (cohort 1) is significantly increased. In detail, the odds ratios for the occurrence of pneumonia in general, atypical pneumonia and IFD were 3.1 (95% CI 1.6–6.1, *p = 0*.*001*), 2.9 (95% CI 1.5–5.7, *p = 0*.*003*) and 3.1 (95% CI 1.5–6.3, *p = 0*.*002*), respectively.

### Analyses of Dectin-1 expression following intensive chemotherapy

**Fig 1:**
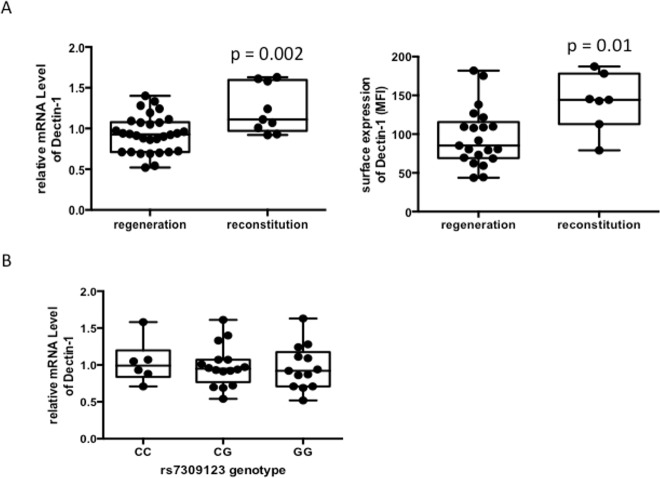
A) Correlation between Dectin-1 mRNA and cell surface expression and time following induction chemotherapy. mRNA levels (left) and surface expression (right) of Dectin-1 measured at two time points following induction chemotherapy: at initial regeneration and at complete reconstitution of blood count. Transcript levels and surface expression were analyzed by quantitative PCR and flow cytometry, respectively on isolated monocytes of AML patients at indicated time points. Shown are the results for Dectin-1 mRNA levels of 30 patients at regeneration and 9 at reconstitution (p = 0.002) and for Dectin-1 surface expression 21 patients at regeneration and 7 at reconstitution (p = 0.01). Y238X carriers were excluded from this analysis. B) No difference in mRNA expression in correlation to Dectin-1 rs7309123 genotype. Expression of Dectin-1 rs7309123 mRNA levels was measured by quantitative PCR. Experiments were performed on isolated monocytes of AML patients. Shown are the results of 6 C/C, 16 C/G and 13 G/G samples, assessed in triplicates.

Since the effectiveness of immune reconstitution following induction chemotherapy is crucial for host defense against fungal infections we analyzed Dectin-1 mRNA expression of CD14^+^ isolated monocytes both during initial hematopoietic regeneration after induction chemotherapy and at complete reconstitution of blood count. mRNA levels were significantly lower during initial hematopoietic regeneration compared to a complete hematopoietic reconstitution prior to consolidation treatment (p = 0.002; Fig 1A). As for Dectin-1 surface expression analysis we excluded Y238X carriers as they are characterized by a reduced cell surface expression (S2 Fig). Consistent with the mRNA results Dectin-1 surface expression was also reduced during initial regeneration compared to a complete blood count reconstitution (p = 0.01; Fig 1A).

To further understand the clinical impact of the Dectin-1 rs7309123 polymorphism we correlated mRNA expression levels of isolated monocytes. Patients harbouring the risk allele G showed comparable Dectin-1 mRNA levels as patients with the C/C (C/G) genotype (Fig 1B).

## Discussion

Here, we report the impact of Dectin-1 and TLR2 polymorphisms on susceptibility to different types of pneumonia including pulmonary IFD in patients receiving induction chemotherapy for acute myeloid leukemia. In our study we could demonstrate a striking correlation between pulmonary infections and individuals that are carriers of the Dectin-1 rs7309123 polymorphism (C/G and G/G). Patients harbouring these genotypes had a two to three times increased risk of pneumonia (including IFD) following induction chemotherapy. Due to the low rate of the T/T carrier status of the Dectin-1 SNP rs3901533 statistics for this SNP were only of descriptive manner. In contrast to other studies, no correlation between the Dectin-1 Y238X status and susceptibility to IFD in our cohort was found in our cohort of patients. The Dectin-1 SNP rs16910525 coding for an early stop codon (Y238X) is functionally well studied and has been linked to the occurence of invasive aspergillosis (IA) in patients after allogeneic transplantation [20]. Two other intronically located SNPs of Dectin-1 (rs3901533 and rs7309123) might also increase the susceptibility to IA after allogeneic stem cell transplantation [12]. The TLR2 SNP rs5743708 coding for an amino acid change R753Q has been linked to increased incidence of tuberculosis, cytomegalovirus (CMV)—disease and other infectious diseases but no association to IFD is known to date [21, 22]. Although minimal changes in cell surface expression of wild type and mutant TLR2 receptor have been described they probably do not contribute to signaling deficiency. Instead, biochemical assays demonstrated that the amino acid change R753Q renders TLR2 signaling by impairing its tyrosine phosphorylation, dimerization with TLR6 and recruitment of Mal and MyD88 [23].

Importantly, all allele frequencies in our report are in concordance with findings from previous studies [12, 24].

Several studies have shown that Dectin-1 and TLR2 act synergistically in recognition and initiation of clearing microbes [13]. Dectin-1 expression enhances TLR2- mediated activation of NF-κB and they collaborate in mediating production of cytokines like interleukins and tumor necrosis factor in response to ß-glucan containing particles [25]. Furthermore, TLR2 is involved in the activation of macrophages that has been described in response to *Aspergillus fumigatus* and *Candida albicans* [26]. The cooperation between Dectin-1 and TLR2 has also been shown following mycobacterial infection [27].

The most extensively studied functional polymorphism within the Dectin-1 gene is the Y238X SNP resulting in an early stop codon. Functional relevance of Dectin-1 loss on the cell surface has been shown in individuals carrying homozygous polymorphism of Y238X who developed recurrent vulvovaginal candidiasis [11]. Another study showed an increased incidence of gastrointestinal Candida colonization in HSCT recipients that were heterozygous for Y238X [28]. Conflicting data are published on the impact of the Dectin-1 SNP Y238X and the association with IFD in patients with haematological malignancies. In hematopoetic stem cell recipients Cunha et al. could demonstrate that the presence of Y238X is associated with an increased susceptibility to aspergillosis irrespective if the donor or the recipient was carrier of the polymorphism [20]. In contrast, Chai et al. could not show a significant association between the Y238X polymorphism and IA in a comparable clinical setting [29]. In our study on we could not replicate a clinical relevance of the Y238X genotype status for the susceptibility to IFD. Both Cunha at al. and Chai et al. performed their studies on patients who mostly underwent HSCT. Since both donor and recipient genotype influence the function of the immune cells it needs to be discussed if chimerism is actually achieved even at the level of pulmonary macrophages that present one of the most important cells of host defense against infectious pathogens causing pulmonary infections. In contrast, in our study a homogenous patient cohort was analyzed that received a defined induction chemotherapy regime. In the study of Cunha et al. the donor Y238X polymorphism was associated with IA at 36 months following transplantation and after 6 months only a trend could be detected. Observation time in our study ended with discharge of the hospital so that we might miss potential delayed effects of that polymorphism.

Other polymorphisms within the Dectin-1 gene were associated with IA susceptibility as well. Sainz et al. could demonstrate an association between the Dectin-1 SNPs rs3901533 and rs7309123 and the rate of IA in patients with hematologic diseases while most of the patients in this cohort underwent HSCT [12]. This heterogeneous cohort comprised 182 hematological patients including only 68 AML patients.

In our study we could identify G-allele carriers of the Dectin-1 rs7309123 polymorphism to be at an increased risk of pulmonary infections including pulmonary IFD following induction chemotherapy. Sainz et al. found that patients carrying the G/G genotype showed a decreased level of Dectin-1 mRNA expression compared to individuals harbouring the C allele (C/C and C/G) [12]. Although those experiments were performed on a rather small number of samples (n = 3 individuals for G/G genotype) they speculate that the Dectin-1 rs7309123 G allele may disrupt binding sites for potential transcription factors and further speculate that it might have an effect on subsequent immune responses. However, in our analysis Dectin-1 mRNA expression was demonstrated to be equal between different Dectin-1 rs7309123 genotypes. In contrast to the study of Sainz et al. using total RNA, we performed experiments on isolated monocytes that represent the predominant cell population of Dectin-1 surface expression.

Induction of Dectin-1 mRNA results in the production of IL-6, TNF-α and other agents that participate in host defense [20]. Thus we were also interested in potential differences in mRNA expression levels of individuals in our patient cohort at certain time points following chemotherapy. In detail, we compared Dectin-1 mRNA expression and Dectin-1 cell surface of isolated monocytes during ongoing hematopoietic regeneration and at complete restored blood count to assess further reasons of susceptibility to fungal infections besides polymorphisms of the Dectin-1 gene. Indeed, both Dectin-1 mRNA expression and surface expression of monocytes during hematopoietic regeneration was significantly lower compared to the later time point when the blood count was restored. These data suggest an impaired immune response to fungal pathogens during the time of chemotherapy- induced immunosuppression due to reduced mRNA levels of Dectin-1. Further validation using functional assays is required to support these findings.

As for the TLR2 SNP R753Q which has a prevalence of about 10% in the Caucasian population several studies have reported an association between this polymorphism and the occurrence of infectious events [21, 30]. In this study, the risk of pneumonia and in particular of pulmonary IFD was significantly increased in patients with a TLR2 R753Q polymorphism although we are aware that these results are based on a rather low number of patients heterozygous for that SNP.

In summary, our study represents the first analysis demonstrating that the very frequent G/G and C/G genotypes of the Dectin-1 SNP rs7309123 and the functionally relevant TLR2 SNP R753Q represent important genetic risk factors of developing pneumonia including pulmonary IFD in AML patients undergoing induction chemotherapy. Since we could not reproduce the association of the Dectin-1 Y238X polymorphism and pulmonary IFD it may be considered that the different Dectin-1 SNPs are part of a stronger haplotype and an interaction of different genetic variants may contribute to the susceptibility of infectious events. Gene—gene interactions involving Dectin-1 with CCL2 (CC-chemokine ligand type 2) and CCR2 (CC-chemokine receptor type 2) performed by epistasis analyses suggest the presence of variants that might contribute to the risk of developing IFD [12]. Further studies are needed to demonstrate a broader view of the interaction of different SNPs of the innate immune system and to obtain a more comprehensive picture of the clinical relevance of such polymorphisms.

## Supporting Information

S1 Fig SNP frequency. SNP and genotype frequencies of the TLR2 and Dectin-1 polymorphisms.
(DOCX)

S2 Fig Dectin-1 Y238X polymorphism associates with reduced Dectin-1 cell surface expression. Surface Dectin-1 expression of CD14^+^ monocytes from wildtype (A/A) or heterozygous (A/C) AML patients for the Dectin-1 Y238X polymorphism. Dectin-1 expression was assessed in 26 WT and 8 Y238X heterozygous individuals (p = 0.006). Representative flow cytometry graphs of extracellular Dectin-1 staining are shown.
(DOCX)

S1 Table Multivariate analyses of the attributable risk of the TLR2 and Dectin-1 polymorphisms. Multivariate analyses of the attributable risk of the TLR2 and Dectin-1 polymorphisms. Abbreviations: OR, odds ratio; CI, confidence interval.
(DOCX)
