# Supplementary material for: Polymorphisms of Dectin-1 and TLR2 Predispose to Invasive Fungal Disease in Patients with Acute Myeloid Leukemia
Source: PLoS One. 2016 Mar 10;11(3):e0150632. doi: 10.1371/journal.pone.0150632 (PMC4786091; doi:10.1371/journal.pone.0150632)
Supplement: S1 Fig — SNP and genotype frequencies of the TLR2 and Dectin-1 polymorphisms. (DOCX) [file pone.0150632.s001.docx]

**S1 Fig**: SNP frequency

S1 Fig:

SNP and genotype frequencies of the TLR2 and Dectin-1 polymorphisms
