# Supplementary material for: Polymorphisms of Dectin-1 and TLR2 Predispose to Invasive Fungal Disease in Patients with Acute Myeloid Leukemia
Source: PLoS One. 2016 Mar 10;11(3):e0150632. doi: 10.1371/journal.pone.0150632 (PMC4786091; doi:10.1371/journal.pone.0150632)
Supplement: S2 Fig — Surface Dectin-1 expression of CD14+ monocytes from wildtype (A/A) or heterozygous (A/C) AML patients for the Dectin-1 Y238X polymorphism. Dectin-1 expression was assessed in 26 WT and 8 Y238X heterozygous individuals (p = 0.006). Representative flow cytometry graphs of extracellular Dectin-1 staining are shown. (DOCX) [file pone.0150632.s002.docx]

**S2 Fig:** Dectin-1 Y238X polymorphism associates with reduced Dectin-1 cell surface expression.

.

S2 Fig:

Surface Dectin-1 expression of CD14^+^ monocytes from wildtype (A/A) or heterozygous (A/C) AML patients for the Dectin-1 Y238X polymorphism. Dectin-1 expression was assessed in 26 WT and 8 Y238X heterozygous individuals (p = 0.006). Representative flow cytometry graphs of extracellular Dectin-1 staining are shown.
