# Supplementary material for: Polymorphisms of Dectin-1 and TLR2 Predispose to Invasive Fungal Disease in Patients with Acute Myeloid Leukemia
Source: PLoS One. 2016 Mar 10;11(3):e0150632. doi: 10.1371/journal.pone.0150632 (PMC4786091; doi:10.1371/journal.pone.0150632)
Supplement: S1 Table — Multivariate analyses of the attributable risk of the TLR2 and Dectin-1 polymorphisms. Abbreviations: OR, odds ratio; CI, confidence interval. (DOCX) [file pone.0150632.s003.docx]

**S1 Table:** Multivariate analyses of the attributable risk of the TLR2 and Dectin-1 polymorphisms.

| Genetic variable | *Pulmonary*  *IFD* %* | OR (95% CI)  *P value* |
| --- | --- | --- |
| Dectin-1 C/C + C/G vs. | 2.9% | 4.0 |
| Dectin-1 rs7309123  G/G genotype | 10.6% | (1.0 – 15.6)  0.047 |
| Dectin-1 C/C vs.  Dectin-1 rs7309123  G/G + C/G genotype | 0%  6.8% | 1.4  (1.3 - 1.6)  0.06 |

* including only probable (n = 7) and proven (n = 2) IFD according to the EORTC/MSG criteria

S1 Table:

Multivariate analyses of the attributable risk of the TLR2 and Dectin-1 polymorphisms.

Abbreviations: OR, odds ratio; CI, confidence interval
